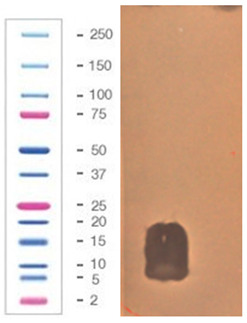# *Paenibacillus alvei MP1* as a Producer of the Proteinaceous Compound with Activity against Important Human Pathogens, Including *Staphylococcus aureus* and *Listeria monocytogenes* †

**DOI:** 10.3390/pathogens9050319

**Published:** 2020-04-25

**Authors:** Magdalena Pajor, Zirui Ray Xiong, Randy W. Worobo, Piotr Szweda

**Affiliations:** 1 Department of Pharmaceutical Technology and Biochemistry, Faculty of Chemistry, Gdansk University of Technology, G. Narutowicza Street 11/12, 80-233 Gdansk, Poland; 2 Department of Food Science, Cornell University, Stocking Hall, Ithaca, New York, NY 14853-7201, USA; zx97@cornell.edu (Z.R.X.); rww8@cornell.edu (R.W.W.)

**Keywords:** *Paenibacillus alvei*, antimicrobial activity, *Staphylococcus aureus*, *Listeria monocytogenes*, *Escherichia coli*, antimicrobial compounds, antimicrobial peptides

## Abstract

An emerging need for new classes of antibiotics is, on the one hand, evident as antimicrobial resistance continues to rise. On the other hand, the awareness of the pros and cons of chemically synthesized compounds’ extensive use leads to a search for new metabolites in already known reservoirs. Previous research showed that *Paenibacillus* strain (*P. alvei* MP1) recovered from a buckwheat honey sample presented a wide spectrum of antimicrobial activity against both Gram-positive and Gram-negative pathogens. Recent investigation has confirmed that *P. alvei* MP1 (deposited at DDBJ/ENA/GenBank under the accession WSQB00000000) produces a proteinaceous, heat-stable compound(s) with the maximum antimicrobial production obtained after 18 h of *P. alvei* MP1 growth in LB medium at 37 °C with continuous shaking at 200 RPM. The highest activity was found in the 40% ammonium sulfate precipitate, with high activity also remaining in the 50% and 60% ammonium sulfate precipitates. Moderate to high antimicrobial activity that is insensitive to proteases or heat treatment, was confirmed against pathogenic bacteria that included *L. monocytogenes* FSL – X1-0001 (strain 10403S), *S. aureus* L1 – 0030 and *E. coli* O157: H7. Further studies, including de novo sequencing of peptides by mass spectrometry, are in progress.

## 1. Introduction

The *Paenibacillus* (the Latin adverb *paene*, meaning almost—almost *Bacillus*) genus are characterized as rod-shaped, aerobic or facultatively anaerobic, and endospore formations. Previously within the *Bacillus* group, *Paenibacillus* was reclassified as a separate genus in 1993. In 1997, Shida et al. [1] proposed to identify members of the *Paenibacillus* cluster and its differentiation from other *Bacillacae* by 16s rRNA gene amplification with purpose-designed primer PAEN515F. The presence of peritrichous flagella in the *Paenibacillus* group was also described. The *Paenibacillus* genus is comprised of approximately 211 [2] to 230 species according to the List of Prokaryotic Names with Standing in Nomenclature [3] with 31 in human samples [4] and 11 newly detected species [2] 

*Paenibacillus* species are ubiquitous and easily isolated from samples with various environmental sources. Recent studies have indicated rhizosphere, soil, and water as the most common reservoirs of *Paenibacillus* representatives. The abilities to promote plant growth, nitrogen fixation, phosphate solubilization, antifungal metabolites production, and overall plant disease prevention are the most outstanding *Paenibacillus* genus features beneficial for agricultural applications [4]. *P. polymyxa* (previously known as *B. polymyxa*) strains are recognized as plant growth promoters for biotechnological purposes, and there is an increasing interest and demand for its industrial production [5,6]. *P. polymyxa* is also known for producing peptide antibiotics—polymyxins [7,8], polypeptins, gavaserin, saltavalin, or jolipeptin*. P. polymyxa* A26 extracellular matrix is an effective antagonist against *Fusarium graminearum*, a predominant causative agent of Fusarium Head Blight (FHB), by producing a mycotoxin known as deoxynivalenol [9]. Furthermore, *Paenibacillus* strains are recognized as producers of antibiotics for medical applications with the potential for treating multidrug-resistant (MDR) human pathogenic bacterial infections [10].

*S. aureus* belongs to the most frequently isolated pathogen in human clinics [11], associated both with skin and respiratory infections and staphylococcal food poisoning (SFP) through enterotoxin production [12]. This bacterium is also notorious for the capacity to acquire resistance to various antibiotic classes [13]. Therefore, new antibacterial agents are required for effective staphylococcal infection treatment. *L. monocytogenes* has a ubiquitous nature, and it is considered as a foodborne pathogen. In terms of cross-contamination, *L. monocytogenes* has the highest mortality rate compared to *Escherichia coli,* and *Salmonella* [14,15]. Naturally occurring antimicrobials, such as bacteriocins, organic acids, essential oils, or chitosan, combined with other stress factors, represent a useful tool against *L. monocytogenes* growth in food [16]. *E. coli* O157: H7 is a common foodborne pathogen that became an increasing public health concern in Europe, as antibiotic resistance is spreading globally [17]. Moreover, the antibiotic treatments for *E. coli* O157: H7 infection may lead to unexpected side effects such as induction of Shiga toxin [18]. 

The strain *P. alvei*, previously isolated from buckwheat honey [19], was found to produce a compound(s) with antimicrobial activity against important human pathogens, including *S. aureus*, *L. monocytogenes*, and *E. coli*. In the present study, strain growth conditions were optimized to achieve maximum antimicrobial production. Partial purification with ammonium sulfate precipitation, solid-phase extraction, and the effect of temperature and enzymes on the active compound was performed to characterize the nature of the antimicrobial compound produced by the strain. 

## 2. Results

### 2.1. Strain Growth Condition Associated with an Active Compound Production

**Figure 1:**
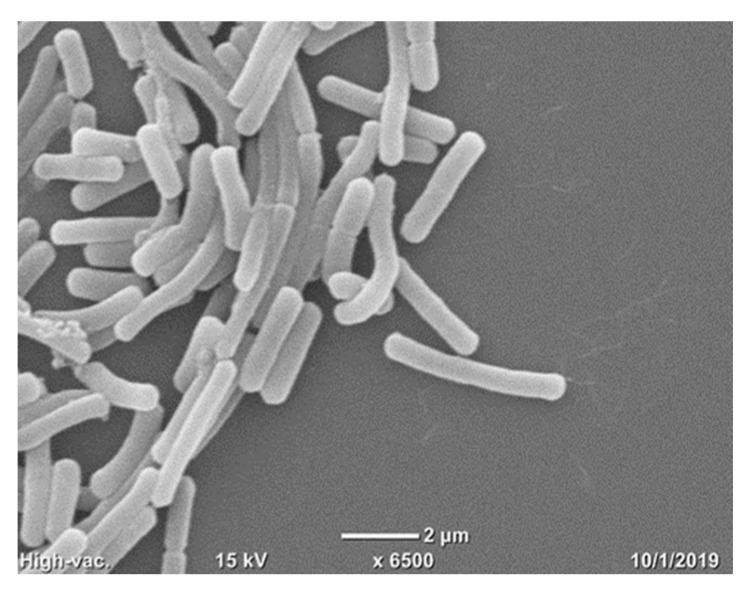
Scanning electron microscope examination of *P. alvei* MP1 cells at a magnification of 6500.

Scanning Electron Microscope (SEM) of the investigated strain is presented in Figure 1. Observation of P. alvei MP1 illustrates rod-shaped bacterial cells with visible flagella. We also observed the ability of P. alvei MP1 to form collective clusters, which might be explained by the production of lubricating fluid for movement on hard surfaces [20].

**Figure 2:**
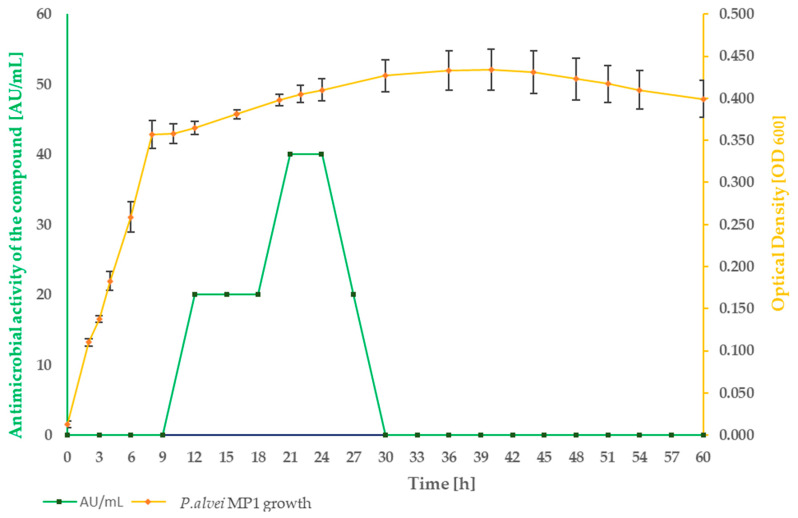
Profile of *P. alvei* MP1 growth and antimicrobial compound production in LB medium at 37 °C, 200 RPM in 3 h intervals during 60 h of incubation.

The optimum growth conditions for the investigated strain were achieved in Luria Bertani Broth (LB) medium at 37 °C with continuous shaking at 200 RPM. Growth of the strain and kinetics of an active compound production is represented in Figure 2. 

The active compound was not produced by P.alvei MP1 during the first nine hours of growth (until the bacteria are in the logarithmic growth phase). Its synthesis was induced after 12 h, with a significant increase in active compound production from 18 to 21 h (in the stationary growth phase). Between 21 to 24 h of P.alvei MP1 growth, the level of antimicrobial compound production remained the same. After 24 h, a notable decrease in antimicrobial compound production was observed with no detectable antimicrobial activity after 30 h of growth. A more detailed analysis of the antibacterial activity of P. alvei MP1 cell-free supernatants against S. aureus L1-0030 was performed as two-fold serial dilutions spotted onto TSA soft agar, with S. aureus as the indicator strain. An arbitrary unit (AU) was defined as the reciprocal of the highest dilution that still produced a detectable zone of inhibition and expressed as AU/mL is represented in Table 1. 

The results confirm that the maximum antimicrobial activity level was reached between 18 and 21 h of strain growth calculated as 40 [AU/mL], and it was two times higher comparing to 15 and 24 h of growth. In the preliminary experiment, the growth of *P.alvei* MP1 in different media was compared—TSB (Tryptic Soy Broth) and BHI (Brain Heart Infusion). The efficiency of the strain growth in the LB medium was notability faster. Thus only LB medium was used for the optimization process of active metabolite production presented in the current study.

### 2.2. Inhibitory Spectrum of P. alvei MP1 Cell-Free Precipitates against Indicator Strains

The antibacterial activity of *P. alvei* MP1 cell-free precipitates with different ammonium sulfate saturation was qualitatively and quantitatively assessed by the presence or absence of inhibition zones as well as the determination of arbitrary unit per milliliter (AU/mL), defined as the reciprocal of the highest dilution showing a clear zone of growth inhibition, Table 2. 

The majority of the active compound was found in the cell-free precipitates with 40%, 50% and 60% saturation of ammonium sulfate. However, the highest antibacterial activity of the precipitate protein/peptide against *S.aureus* was 1280 AU/mL, found in cell-free precipitate with 40% saturation of ammonium sulfate. The antimicrobial activity of the cell-free precipitates was also evaluated against *L.monocytogenes* FSL – X1-0001 and *E. coli* O157: H7, Table 3.

The highest antimicrobial activity was 160 AU/mL for both *E. coli* O157: H7 and *L. moncytogenes* FSL – X1-0001, which suggests a lower level of sensitivity of these bacteria in comparison to *S. aureus*. 

### 2.3. Effects of Enzymes and Temperature on Antimicrobial Activity of P.alvei MP1 Cell-Free Precipitates

The effect of proteases on the antimicrobial activity of *P. alvei* MP1 cell-free precipitates with different ammonium sulfate saturation against *S. aureus* L1-0030 is presented in Table 4. The effect of Proteinase K is presented in Table 5. The temperature stability of *P. alvei* MP1 cell-free precipitates with different ammonium sulfate saturation against *S. aureus* L1-0030 is presented in Table 6. 

The protease and heat treatment studies revealed a lack of effect on the antibacterial activity of the antimicrobial compound produced by the *P. alvei* MP1 strain. This is an important advantage of the compound, with potential applications in antimicrobial protection of thermally processed food or the treatment of human and animal bacterial infections.

### 2.4. Solid Phase Extraction (SPE)

**Figure 3:**
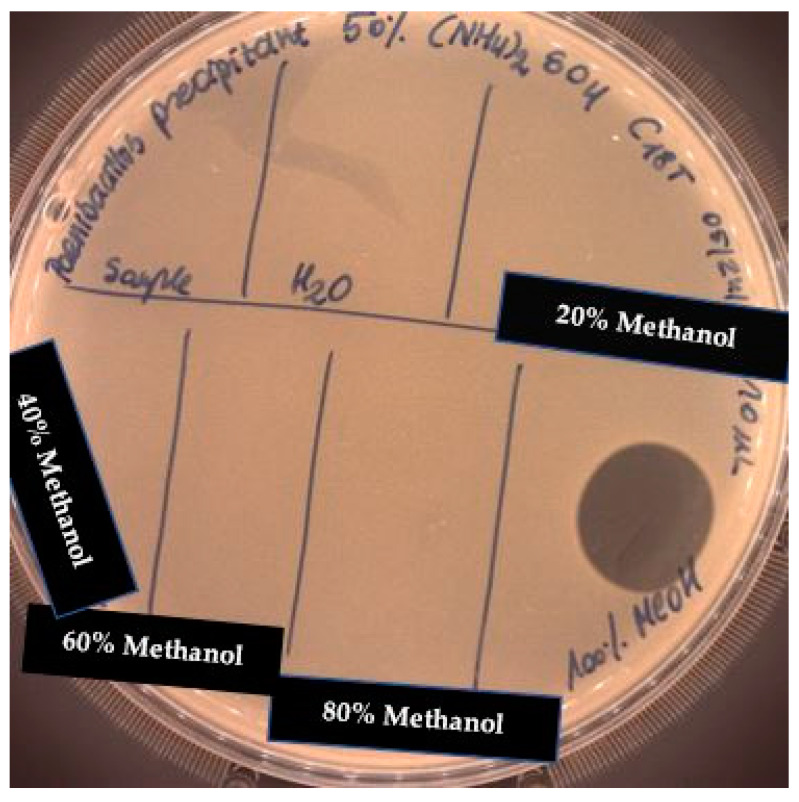
The anti-staphylococcal activity of fractions with increasing concentration of methanol obtained from the SPE purification step.

The SPE method was applied for further purification of the active metabolite of *P. alvei* MP1 using a silica-based cartridge column, Waters Sep-Pak tC18. The metabolites with a growing level of hydrophobicity were eluted from the resin with an increasing concentration of methanol. The optimal concentration of methanol for extracting the antimicrobial activity of interest was 100% (Figure 3). 

**Figure 4:**
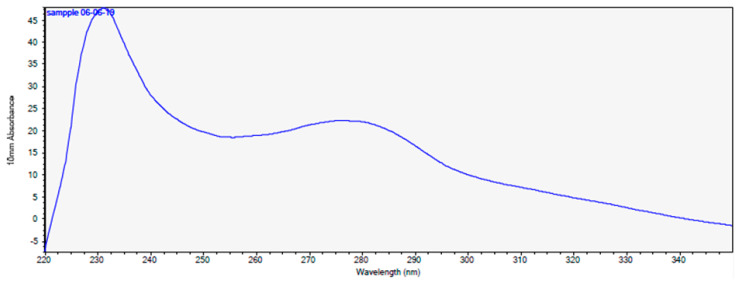
The UV- VIS spectrum after SPE of *P. alvei* MP1 fraction with 50% saturation of ammonium sulfate.

This result suggests the hydrophobic nature of active compounds. Interestingly, the UV-VIS spectrum (Figure 4) of the purified fraction (100% methanol) suggested the proteinaceous nature of the active component, which is surprising in light of the results of the previous assays (treatment with proteases and high temperatures). The theoretical protein concentration in the chosen fraction was 21.786 mg/mL. 

### 2.5. Direct Detection of Antimicrobial Activity on Tricine SDS-PAGE Gels

Two gels were prepared according to the procedure presented in the material and methods Section 4.5. To confirm the proteinaceous nature of the active metabolites, Tricine SDS-PAGE electrophoresis was applied with direct detection of the presence as well as the activity of suspected active components in the structure of the gel. 

**Figure 5:**
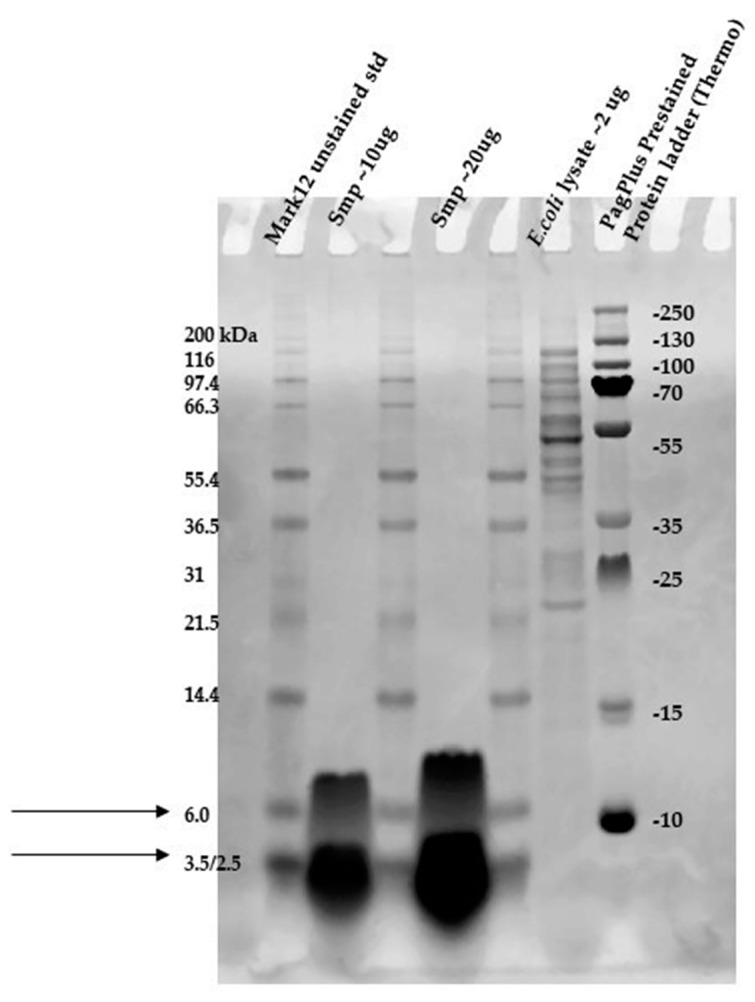
SDS-PAGE of chosen sample purified via SPE (Gel 2).

The gel was prepared in agreement with the standards set by the Cornell Biotechnology Resource Center (BRC) in a 12% Bis-Tris gel/ MES buffer system/ colloidal Coomassie stain presented in Section 4.5 (material and methods). The samples of 10 (line 2) and 20 µg (line 4) of protein that was present in the active fraction from SPE (100% of methanol) was subject of SDS – PAGE, Figure 5. Two standards—Mark12 Unstained Standard consisting of 12 polypeptides in the range of 2.5–200 kDa (lines 1, 3 and 5), and PageRuler Plus Prestained Protein in the range of 10 to 250 kDa (line 7)—were used for molecular weight estimation. The lysate of *E.coli ~* 2 µg was loaded for comparison (line 6).

In the gel, we observed two interesting bands (in lines 2 and 4) corresponding to the proteins/peptides with molecular weights ranging from 3.5 to 6 kDa according to Mark12 Unstained Standard. This result could suggest the coexistence of two active compounds or partial hydrolysis of the active compound and, first of all, finally confirmed the proteinaceous nature of this compound.

The antimicrobial activity of the sample after SDS–PAGE was evaluated against three bacterial indicator strains, Table 7; the assay was performed according to the methodology presented in Section 4.5 (material and methods).

The chosen sample exhibited a wide spectrum of antimicrobial activity. However, results indicated that *S. aureus* L1 – 0030 was the most sensitive strain, which is confirmed by the fact that the growth inhibition zone for this strain was visible even after 24 h of incubation. In the case of two other strains, *L. monocytogenes* FSL – X1 – 0001 and *E. coli* O157: H7, respectively, the growth inhibition zones were visible only at the early stages of bacteria growth within the structure of agar medium (up to 7–12 h). It can be assumed that in the volume of the agar where the active component was present (as a result of diffusion from the gel), the process of bacterial cell dividing was partially inhibited (but not completely inhibited) and not all bacteria were killed. As a result of longer incubation, the growth inhibition zones for the aforementioned strains disappeared. In our opinion, results with this assay using Tricine SDS-PAGE gels (observation of growth inhibition zones) finally confirm the proteinaceous nature of the active agent produced by the *P. alvei* MP1 isolate. However, it is resistant to the activity of several proteolytic enzymes (including Proteinase K) and also heat treatment.

### 2.6. High-Performance Liquid Chromatography (HPLC)

**Figure 6:**
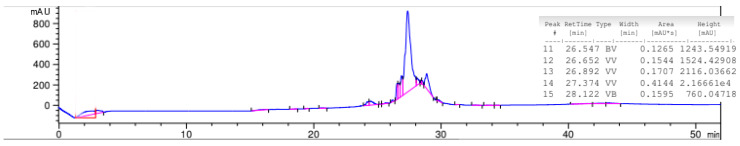
Peaks of interest with column effluent monitored at 216 nm.

The total number of peaks of interest monitored with column effluent monitored at 216 nm is presented in Figure 6.

Three fractions with retention times as follow: 27.0–27.7 (fraction 1), 27.7–28.5 (fraction 2) and 28.5–29.5 min (fraction 3), were collected. 

**Figure 7:**
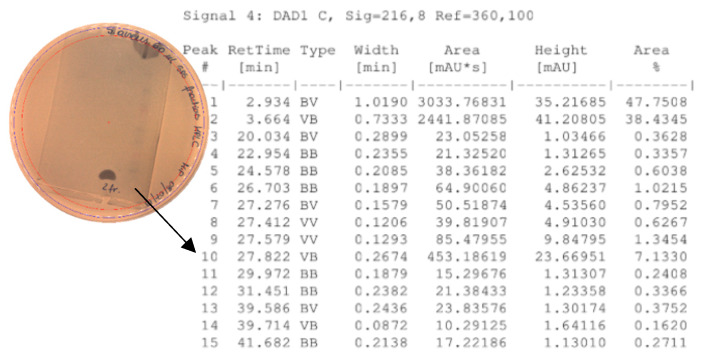
Agar-overlay inhibition assay with SDS-PAGE gel of collected fraction with the retention time of 27.82 min against *S. aureus* L1-0030.

The acetonitrile in the samples was evaporated under vacuum. An agar-overlay inhibition assay with SDS-PAGE gel of the collected fractions against *S. aureus* L1-0030 was performed. Fraction 2 with the retention time of 27.82 min and a peak of 23.67 mAU exhibited antibacterial activity on SDS–PAGE gel in the total loading volume of 4 µL (total 1:3 ratios with buffer) against the most sensitive strain *S. aureus L1-0030*, Figure 7.

This fraction was the subject of our further research aiming in the final characterization of the active component produced by *P. alvei* MP1 strain. 

## 3. Discussion

Several publications have confirmed that microorganisms naturally occurring in the environment and food are an abundant source of compounds with antimicrobial activity [21,22,23,24].

Recent studies show that, despite a negative opinion about *Paenibacillus* group members, mainly as a result of *P. larvae* being recognized as the causative agent of American Foulbrood, bacteria belonging to this genus are a rich source of metabolites with promising antimicrobial activity [25,26,27]. *P. alvei* AN5 [28] was detected as a producer of an antimicrobial compound against both Gram-positive and Gram-negative bacterial strains, including *S. aureus* and *E. coli* ATCC 29522. The compound subjected to SDS–PAGE was also characterized as proteinaceous, similarly as the active substance produced by *P. alvei* MP1 investigated in this study. 

Two different antimicrobial peptides characterized as Paenibacillin N and P with potent antimicrobial activity against many clinical pathogens were produced by *P. alvei* NP75 [29]. Interestingly, compounds were differentially synthesized—Paenibacillin N was non-ribosomally synthesized, whereas Paenibacillin P was synthesized ribosomally. Moreover, the aforementioned strain produced an extracellular protease defending itself from Paenibacillin P. 

Discovered to date, *Paenibacillus* species are producers of two of the three classes of bacteriocins, being lantibiotics and pediocins, whereas the search within the group of pediocins is less extensive. The lantibiotic, called Paenibacillin, presented a wide range of antimicrobial activity, and heat stability was first reported in 2007 [4]. The investigation of recently discovered Paenibacillin discovered from *P. polymyxa* OSY – DF revealed unique features in its biosynthesis [30]. The authors determined that the production of Paenibacillin is eliminated by the disruption of the gene PaeB, encoding for lantibiotic dehydratase.

Bacteria of the genus *Paenibacillus* are also known to produce several peptides (exhibiting antimicrobial activity), which are not ribosomally synthesized and do not belong to bacteriocins, but they are classified as secondary metabolites. Among them, lipopeptides are particularly attractive to medical applications as antimicrobial agents. The enzymes, called Non-Ribosomal Peptide Synthetases (NRPS) [10,28], are crucial for their production in bacterial cells. Lipopeptides produced by *Paenibacillus* species were in 2016 named by Cochrane et al. [10] as the “gold mine for antibiotic candidates.” Polymyxins are so far the most studied of them and constitute about 15 variants [31]. They also represent the oldest known group of lipopeptides after their discovery in 1947. The antimicrobial and antifungal activity of lipopeptides varies according to the lipid group length and carbon atom content [32]. According to Wu et al. [33] and Qian et al. [34], residues from di-amino butyric acid (Dab) and a C6-C7 N-terminal fatty acyl chain are at a high percentage in most of the described lipopeptide antibiotics within the *Paenibacillus* group. Variations within the peptide composition, the polar head, or the fatty acid tail with different degrees of branching and oxidation lead to the structural diversity and complexity of the lipopeptides [35].

The agent produced by *P. alvei* MP1 also seems to be very hydrophobic—it was eluted from the chromatography column (SPE step of purification) with the mobile phases composed of only methanol. It can suggest that the agent of interest also belongs to lipopeptides. Moreover, SDS-PAGE analysis revealed the existence of two substances in active fractions (two bands on the gels are observed). These may suggest two variants of the agent lipopeptide, with and without the lipid moiety. Determining the exact nature and structure of the active metabolite of *P. alvei* MP1 is the subject of our current investigation.

A broad range of the spectrum, high thermal stability as well as resistance to the activity of proteases of high proteolytic activity (including proteinase K which can digest keratine—a component of hairs and nails) should be considered as important advantages of the investigated agent from the point of view of possibilities of its application for antimicrobial protection of food products and/or clinical settings. The newly described metabolite of *P. alvei* MP1 exhibited considerable activity against all three species of bacteria used as indicatory strains, namely *E. coli*, *L. monocytogenes*, and *S. aureus*. All of them are classified as both important human and animal pathogens and leading etiological factors of bacterial foodborne illness (foodborne infections, in the case of *E. coli* and *L. monocytogenes* and toxoinfections in the case of *S. aureus*). *S. aureus*, which exhibited the highest susceptibility, is recognized as pathogenic bacteria that quickly develop mechanisms of resistance to a plethora of antibiotics currently used in clinical practice. Thus new agents effective against these bacteria are urgently needed. Because of high resistance to temperature and proteolysis, the *P. alvei* MP1 metabolite could be used for the elimination of pathogenic bacteria from food raw materials that contain proteases (e.g., meat or fish) and undergo thermal processing with relatively high temperatures of about 100 °C.

As the development of an innovative strategy for antimicrobial-resistant microorganisms remains inevitable, many articles present species of *Paenibacillus* as a source of new compounds with antimicrobial activity that are not covered with currently existing mechanisms of resistance. New antimicrobial compounds produced by food and environment-associated microorganisms were the aim of the research by Gao et al. [5] and Huang et al. [36]. As a result, two members of the *Paenibacillus* group were isolated. A new strain of *Paenibacillus* -P. OSY-SE was isolated from soil and exhibited activity against Gram-positive and Gram-negative bacteria, including *L. monocytogenes*, and *E. coli* O157: H7. The active compound was determined to be a cyclic lipopeptide, Paenibacterin. Huang et al. [37] also isolated two cyclic lipopeptides antibiotics from *Paenibacillus* strain (named B7) isolated from dairy waste that was found to produce antimicrobial agents active against *S. aureus*, *E. coli,* and pan-drug-resistant *P. aeruginosa* clinical isolate. The isolated rod-shaped, spore-forming, motile, Gram-positive strain was identified after the DNA–DNA hybridization as a member of *P. ehimensis*. The researchers highlighted the importance of further studying of these two peptides.

An innovative strategy for novel antimicrobial search should certainly take into account the genomic information of bacteria. Within the *Paenibacillus* group, the genomic information remains insufficient [38]. The potential of *Paenibacillus* spp. to produce a variety of compounds are identified in the previously known clusters; however, discovering new clusters such as the novel lasso peptide tailored by a new class of kinases in *P. dendritiformis* C454 suggests that several antimicrobial compounds of potential use are yet to be characterized [39].

Further studies, including de novo sequencing of peptides produced by *P. alvei* strain MP1 by mass spectrometry, are in progress.

The findings of our previous investigation [19], as well as the results presented by other authors [40,41], revealed that bacteria isolated from honey show the ability to produce a wide range of antimicrobial metabolites. All these producing strains were likely brought to the hive with nectar or pollen that have been collected by bees from flowers of various species. Plants, like other living organisms, have developed numerous mechanisms of protection against the invasion of pathogenic microorganisms, mainly bacteria. Among them, production of different plant antimicrobial peptides (e.g., thionins, defensins, cyclotides [42,43]), and secondary metabolites of plants including terpenoids (polymeric isoprene derivatives and biosynthesized from acetate via the mevalonic acid pathway), phenolics (biosynthesized from shikimate pathways, containing one or more hydroxylated aromatic ring), and alkaloids (non-protein nitrogen-containing compounds, biosynthesized from amino acids, such as tyrosine) are recognized as the most important [44,45]. Besides the plant surface, plant tissues are also inhabited by bacteria and fungi (natural, beneficial microflora) that produce a broad spectrum of antimicrobial agents to protect the host plant against infection [46]. Most of these bacteria, when transferred to the hive, are not able to survive in the environment of matured honey primarily because of high sugar content (about 80%) and low pH (around 4.0). Most of the producing strains isolated by our group and other authors belong to spore-forming bacteria, mainly to the spore-forming bacteria of the genus *Bacillus* spp. that were not killed by harsh conditions of mature honey. Therefore, most of the bacterial strains isolated from honey as producers of antimicrobial substances were classified as *Bacillus* spp. [19,40,41,46]. In our opinion, honey, but also pollen and bee bread, deserve more attention as a potential reservoir of bacteria interesting for both pharmaceutical and biotechnological applications.

## 4. Materials and Methods 

### 4.1. Strain Growth Conditions Associated with an Active Compound Production

Optimization of the growth conditions of P. alvei MP1 was evaluated using commercially available liquid broths—*Luria Bertani Broth* (LB), Tryptic Soy Broth (TSB), and Brain Heart Infusion (BHI) (Becton, Dickinson, and Company (BD), USA). The period of incubation varied from 0 to 60 h with a temperature of 30 and 37 °C. This preliminary investigation revealed the highest level of antimicrobial activity in LB medium at 37 °C at 200 RPM between 18–21 h of growth. Only this medium was used for further investigation. 

*P. alvei* MP1 cells were also examined using a Scanning Electron Microscope (SEM) according to the following protocol [47], except for 5% glutaraldehyde used instead of Karnovsk’s fixative in step 2.

The second objective was to establish the lowest concentration expressed in terms of arbitrary units per milliliter (AU/mL) of the serially diluted *P. alvei* MP1 cell-free supernatant and precipitates against the most sensitive strain, at which bacterial growth is still inhibited. One arbitrary unit (AU) against an individual indicator strain was defined as the reciprocal of the highest dilution that still produced a minimal but detectable zone of inhibition and expressed as AU/mL. Flasks containing 150 mL of LB broth were inoculated with 1.5 mL of *P. alvei* MP1 pre-culture inoculum and were incubated in a shaker at 200 RPM with continuous shaking. The kinetics of antibacterial production was conducted at 37 °C for 60 h in LB medium. The growth was followed by measuring the optical density at 600 nm (OD600). Samples were withdrawn at desired time intervals by centrifugation at 12,000 × g for 15 min, and the supernatants and precipitates were tested for an active compound activity against indicator strains. Antimicrobial activity was initially assessed by direct spotting on an agar plate and was performed multiple times during the current study. Briefly, 50 μL of tested substance was aseptically spotted on the surface of the LA plate and air-dried aseptically in a biosafety cabinet. Next, 7 mL of soft agar (0.75% agar, w/v) with the suspension of 70 µL of an overnight culture of the indicator strain was overlaid on the surface of the plate and allowed to dry. The plates were incubated for 24 h at 37 °C. The presence of inhibition zones was observed, and the results were reported in AU/mL.

### 4.2. Partial Purification of the Bioactive Compound with Ammonium Sulfate

*P. alvei* MP1 strain was grown in 150 mL of LB medium at 37 °C in a rotary shaker at 200 RPM for 18 h. Cells were removed by centrifugation at 12,000 RPM for 20 min. The supernatant was heated at 72 °C for 30 min to inactivate bacterial cells, then chilled to 4 °C and sequentially precipitated by stepwise addition of solid ammonium sulfate of saturation degree as follows: 20%, 40%, 50%, 60%, 70% and 80% with continuous stirring at 4 °C for 24 h. The precipitated proteins were collected by centrifugation (12,000 RPM for 20 min) and dissolved in dH2O sufficient to dissolve it completely. Aliquots of precipitated fractions were analyzed for their protein concentration and antimicrobial activity. 

### 4.3. Effects of Proteolytic enzymes and Temperature on Antimicrobial Activity of the Compound

The compound produced by *P. alvei* MP1 strain was assessed for its sensitivity to proteases— Pepsin, Chymotrypsin, Pronase E, and Proteinase K (Sigma-Aldrich, St. Louis, MO) and its thermal stability. Fifty microliters of a *P.alvei* MP1 cell-free culture with different ammonium sulfate saturation was sterilely spotted on the surface of the LA plate and air-dried aseptically in a biosafety cabinet. Next, 10 μL or 5 μL of proteinases were spotted and allowed to dry. Next, 7 mL of soft agar (0.75% agar, w/v) with the suspension of 70 µL of an overnight culture of the indicator strain was overlaid on the surface of the plate and allowed to dry. The plates were incubated for 24 h at 37 °C. The presence of inhibition zones was observed. The antibacterial activity was determined against *S. aureus* L1-0030. Untreated samples, buffer, and enzyme solutions served as controls.

To analyze thermal stability, aliquots of active compounds were incubated at different temperatures in the range of 60, 80, and 100 °C for 30 min. After cooling to room temperature, antimicrobial activity against *S. aureus* L1-0030 was determined according to the method described above.

### 4.4. Solid-Phase Extraction with tC18 Column

Solid-phase extraction was carried out with a Sep-Pak tC18 column (Waters) with methanol as a solvent in gradient concentration as follows: 0% (control sample), 20%, 40%, 60%, 80% and 100% from *P. alvei* MP1 precipitates with demonstrated antimicrobial activities. The extracted phases were evaporated from methanol residues using vacufuge at 45 °C for 1 h. After evaporation, the pellet was dissolved in dH2O, and theoretical protein concentration was measured on NanoDrop (Thermo Fischer Scientific, USA). The antimicrobial activity of collected extracts against *S. aureus* L1-0030 was assessed. 

### 4.5. Agar-Overlay Inhibition Assay on SDS-PAGE Gels

Two SDS-PAGE gels were prepared as follows:

Gel 1: This gel was used for direct detection of the antimicrobial activity against *L. monocytogenes* FSL – X1 – 0001, *S. aureus* L1 – 0030, and *E. coli* O157: H7 and was washed with distilled water after fixation step. Eight microliters of the sample with a final concentration of 5.49 mg/mL and 3 µL of molecular-weight standard (Bio-Rad Precision Plus Protein™ Dual Color Standards – Bio-Rad, USA) ranging from 10 to 250 kDa were loaded to Mini-PROTEAN precast gels for polyacrylamide gel electrophoresis (PAGE) (Bio-Rad Mini-PROTEAN® TGX Stain-Free™ Precast Gels, Bio-Rad, USA). Electrophoresis conditions and protein visualization with Coomassie Blue Staining were conducted according to the following protocol [48]. 

Gel 2: The gel was prepared for band visualization in agreement with the standards set by the Cornell Biotechnology Resource Center (BRC) in 12% Bis-Tris gel/ MES buffer system/ colloidal Coomassie stain.

### 4.6. High-Performance Liquid Chromatography (HPLC)

HPLC was performed in the Food Science Department, Cornell AgriTech Geneva, Cornell University. Samples with the highest antimicrobial activity after SPE were selected for HPLC analysis with fraction collection and evaluation of antimicrobial activity against *S. aureus* L1-0030. The presence or absence of growth inhibition zones was recorded. Next, active fractions with the highest concentration (measured on NanoDrop) were chosen for the second round of HPLC to confirm its purity.

The column was Varian LiChrosper C18 (Agilent Technologies, Santa Clara, CA), (Diameter 4.6 mm, Length 250.0 mm, Particle Size 5 µm). The elution condition was 0–10 min mobile phase A (0.05% TFA in water); 10–40 min a gradient of 0–100% mobile phase B (acetonitrile + 0.05% TFA); and 40–50 min mobile phase B with a flow rate of 1 mL/min. The injection volume was 10 µL. All HPLC solvents were prepared fresh daily, and all aqueous solutions were prepared with ultrapure water. The column effluent was monitored at 216, 218, 220, and 280 nm. Collected fractions were checked for antimicrobial activity against the most sensitive strain.

## 5. Conclusions

Our previous studies revealed the high antimicrobial potential of bee products collected in Polish apiaries [49,50,51]. The current research confirms that honey also should be considered as a reliable source of bacteria producing new antimicrobial agents with promising activity. The *P. alvei* MP1 strain isolated from buckwheat honey produces metabolite or metabolites of proteinaceous nature active against important human pathogens, with particularly high activity against *S. aureus*. The conditions of efficient production of the active compound, as well as an easy method of its purification, have been developed. The research aiming in the determination of the chemical structure of this agent is a subject of our current research. 

## Figures and Tables

**Table 1:** Antibacterial activity of *P. alvei* MP1 cell-free supernatant against *S. aureus* L1-0030. The diameter of each inhibition zone was measured.

Hours of Experiment [h]	Two-fold Serial Dilutions of Cell-Free Supernatant Spotted onto TSA Soft Agar, with *S. aureus* L1- 0030 as Indicator Strain	Antibacterial Activity [AU/mL]	Inhibition Zones Diameter Ø [cm]
9	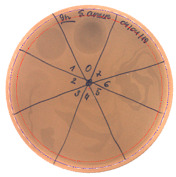	0	-
12	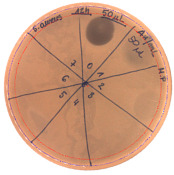	20	1.9
15	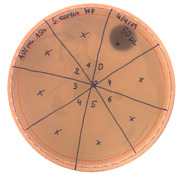	20	1.7
18	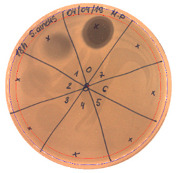	20	1.9
21	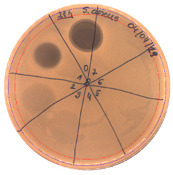	40	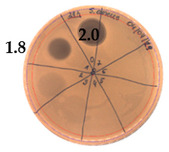
24	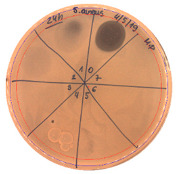	20	2.0
27	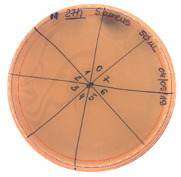	-	-
30	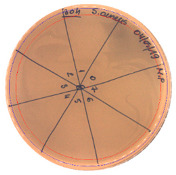	-	-

**Table 2:** Antibacterial potential of *P. alvei* MP1 cell-free precipitates with different ammonium sulfate saturation against *S. aureus* L1-0030 (the two-fold serial dilutions of solutions of precipitates were spotted on agar medium in a clockwise direction. The diameter of each inhibition zone was measured).

Two-fold Serial Dilutions – 50 µL Spots	Inhibition Zone Diameter Ø [cm]
**20% (NH_4_)_2_SO_4_**	
** 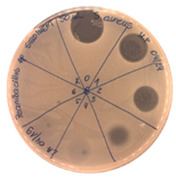 **	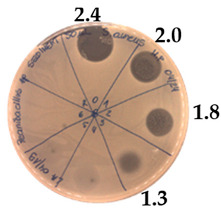
Precipitate Activity—160 AU/mL	
**40% (NH_4_)_2_SO_4_**	
** 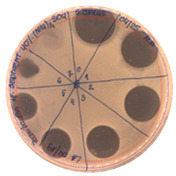 **	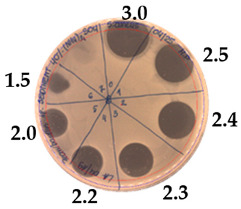
Precipitate Activity—1280 AU/mL	
**50% (NH_4_)_2_SO_4_**	
** 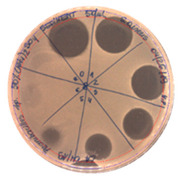 **	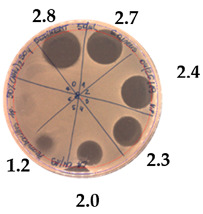
Precipitate Activity—640 AU/mL	
**60% (NH_4_)_2_SO_4_**	
** 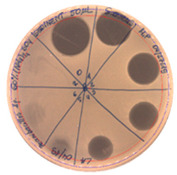 **	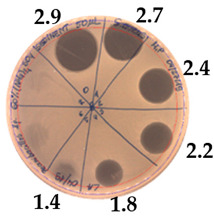
Precipitate Activity—640 AU/mL	
**70% (NH_4_)_2_SO_4_**	
** 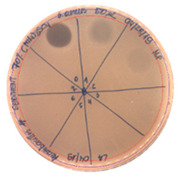 **	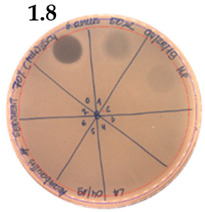
Precipitate Activity—20 AU/mL	

**Table 3:** The antimicrobial potential of *P.alvei* MP1 cell-free precipitates with 40% saturation of ammonium sulfate evaluated against *L.monocytogenes* FSL – X1-0001 and *E. coli* O157: H7 after 24 h of incubation. The diameter of each inhibition zone was measured.

Indicator Strain	
**Two-fold serial dilutions – 50 µL spots**	
*L. monocytogenes* FSL – X1-0001	*E. coli* O157: H7
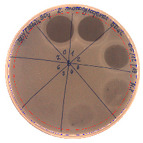	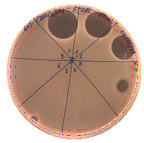
(160 AU/mL)	(160 AU/mL)
**Inhibition zone diameter Ø [cm]**	
*L. monocytogenes* FSL – X1-0001	*E. coli* O157: H7
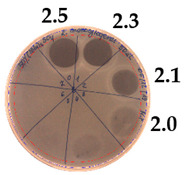	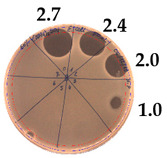

**Table 4:** The effects of proteases on antimicrobial activity of *P.alvei* MP1 cell-free precipitates with different ammonium sulfate saturation against *S. aureus* L1-0030.

Ammonium Sulfate Saturation [%]	Indicator Strain - *S. aureus* L1- 0030
	Two-fold Serial Dilutions – 50 µL Spots
40	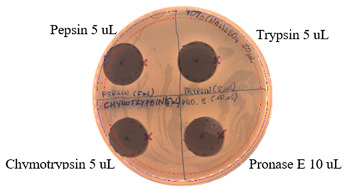
50	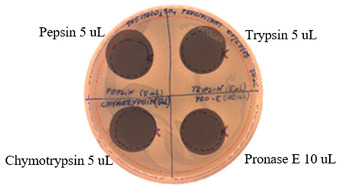
60	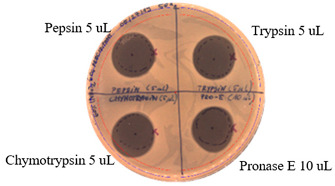

**Table 5:** The effects of proteinase K on antimicrobial activity of *P.alvei* MP1 cell-free precipitates with different ammonium sulfate saturation against *S. aureus* L1-0030.

Proteinase K Test
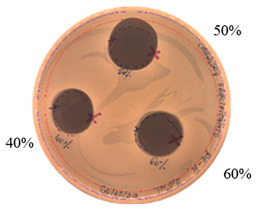

**Table 6:** Temperature stability of *P.alvei* MP1 cell-free precipitates with different ammonium sulfate saturation against *S. aureus* L1-0030.

60 °C for 30 min.	80 °C for 30 min	100 °C for 30 min
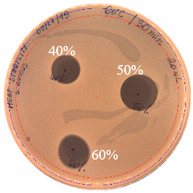	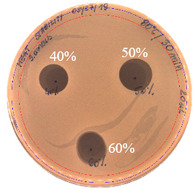	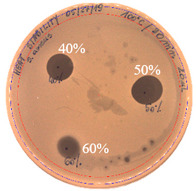

**Table 7:** Inhibition zones observed on the SDS-GELS against bacterial indicator strains after seven and 24 h of incubation (Gel 1 - 8 μL of the samples with a final concentration of 5.49 mg/mL and Bio-Rad Precision Plus Protein™ Dual Color Standards ranging from 10 to 250 kDa).

Incubation Time [h]	Indicator Strain		
	*L. monocytogenes* FSL – X1 - 0001	*S. aureus* L1 - 0030	*E. coli* O157: H7
**7**	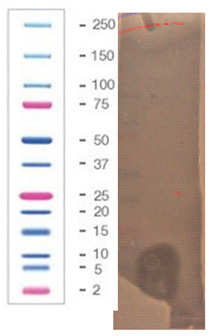	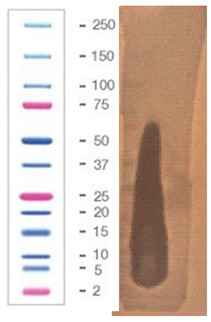	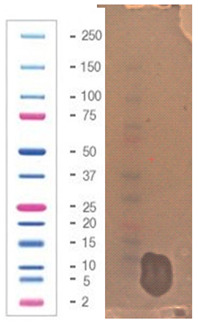
**24**